# Quantum holography with undetected light

**DOI:** 10.1126/sciadv.abl4301

**Published:** 2022-01-14

**Authors:** Sebastian Töpfer, Marta Gilaberte Basset, Jorge Fuenzalida, Fabian Steinlechner, Juan P. Torres, Markus Gräfe

**Affiliations:** 1 Fraunhofer Institute for Applied Optics and Precision Engineering IOF, Albert-Einstein-Str. 7, 07745, Jena, Germany.; 2 Friedrich Schiller University Jena, Institute of Applied Physics, Abbe Center of Photonics, Albert-Einstein-Str. 6, 07745 Jena, Germany.; 3 ICFO-Institut de Ciencies Fotoniques, The Barcelona Institute of Science and Technology, 08860 Castelldefels, Spain.; 4 Department of Signal Theory and Communications, Universitat Politecnica de Catalunya, 08034 Barcelona, Spain.

## Abstract

Holography exploits the interference of a light field reflected/transmitted from an object with a reference beam to obtain a reconstruction of the spatial shape of the object. Classical holography techniques have been very successful in diverse areas such as microscopy, manufacturing technology, and basic science. However, detection constraints for wavelengths outside the visible range restrict the applications for imaging and sensing in general. For overcoming these detection limitations, we implement phase-shifting holography with nonclassical states of light, where we exploit quantum interference between two-photon probability amplitudes in a nonlinear interferometer. We demonstrate that it allows retrieving the spatial shape (amplitude and phase) of the photons transmitted/reflected from the object and thus obtaining an image of the object despite those photons are never detected. Moreover, there is no need to use a well-characterized reference beam, since the two-photon scheme already makes use of one of the photons as reference for holography.

## Teaser

Experiments show the performance of quantum holography using undetected photons.

## INTRODUCTION

In the past few decades, scientists and engineers throughout the world, from different disciplines, governments, and information technology companies, are paying increasing attention to quantum technologies. Quantum communications (*1*–*3*), quantum computation (*4*), and, particularly, quantum imaging (*5*–*9*) are just some examples of novel areas of science and technology where quantum ideas are helping to implement systems with enabling new capabilities. Quantum technologies promise to go further than classical counterpart technologies by using new quantum states of light and matter, performing tasks that are impossible to implement classically (*10*). A clear example of this is the ability to obtain a hologram from single photons (*11*) and even recording the hologram without detecting the photons themselves as we report here.

Holography was introduced by Gabor in 1948 (*12*). It allows the reconstruction of the spatial structure of an object by recording amplitude and phase information of the light reflected from an object. Classical holography is already successfully applied in sensing and microscopy. This is true especially for biospecimen, where scattering and absorption require a phase-sensitive sensing contrast agent-free approach (*13*, *14*). Holography can also be applied in optical security (*15*, *16*) and data storage (*17*, *18*). The introduction of single-photon holographic methods would expand holography to several applications that raised together with the recent growth of quantum technologies (*11*). In particular, single-photon holography with undetected light in a nonlinear interferometer scheme (*19*–*22*) would introduce the benefit of choosing the most convenient spectral range for the probing beam in an application without facing the limitations imposed by the low efficiency of detectors at those specific spectral range (e.g., in the mid-infrared). Nonlinear interferometry has been proven to be key elements in numerous applications, namely, in imaging (*6*, *23*, *24*), sensing (*25*), spectroscopy (*7*, *26*), microscopy (*27*, *28*), and optical coherence tomography (*26*, *29*–*31*).

To characterize the spatial shape of the single photon, we use phase-shifting holography, introduced in 1997 by Yamaguchi and Zhang (*32*), where several images with different phase steps are recorded and processed. This technique allows the reconstruction of the spatial structure (amplitude and phase) of the light reflected from a sample. In combination with the use of the effect of induced coherence (*20*, *21*), we can obtain a hologram without detecting the light that illuminated the sample. Our experiment is a step forward to enable efficient holography in a broader spectral range.

## RESULTS

### Phase-shifting holography with a SU(1,1) nonlinear interferometer

In this part, we give a brief overview of the theoretical framework of phase-shifting holography in the quantum regime considered here. An extended and more detailed theoretical description of the experiment can be found in the Supplementary Materials.

**Fig. 1.**
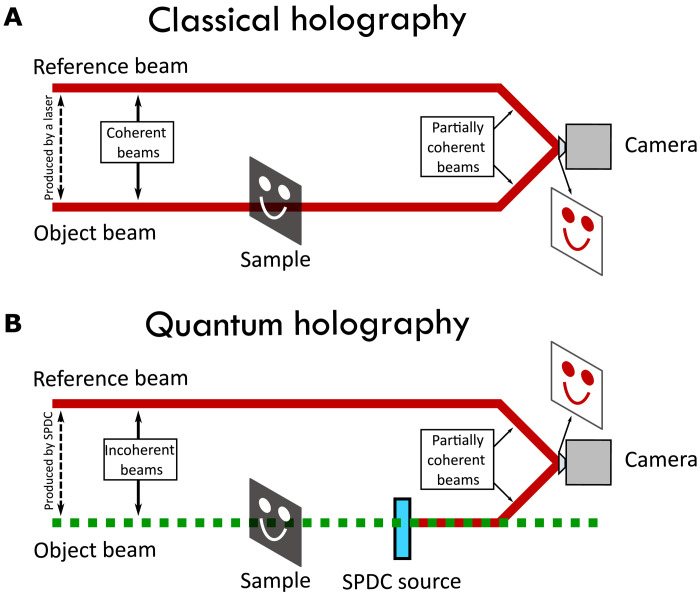
Classical and quantum holography. (**A**) In classical holography, the spatially dependent interference pattern of two coherent beams, the reference beam and the object beam, after interaction with the object, are recorded and used to construct the hologram of the object. (**B**) In our quantum holography scheme, we make use of two-photon states that can be generated by SPDC in one of two sources. The spatial shape of the object, which is transferred to the spatial shape of the light reflected/transmitted from the object, is contained in the probability amplitudes corresponding to the paired photons being generated in either of two SPDC sources.

In classical holography (see Fig. 1A), the hologram is the result of the interference of two input beams with mutual coherence (*33*). One beam serves as reference beam. The other beam (object beam) illuminates an object and generates reflected/transmitted light that bears information of the spatial structure of the object. The spatial shape of the interference pattern, resulting from combining the object and reference beams

$$I(x,y)=I_{r}(x,y)+I_{o}(x,y)+2\sqrt{I_{r}(x,y)I_{o}(x,y)}\text{cos}\;[\theta_{o}(x,y)-\theta_{r}(x,y)]$$ (1)

is recorded in the hologram and can be visualized with the help of a charge-coupled device camera (*33*). Here, *I*_r_ and θ_r_(*x*, *y*) are the intensity and phase of the reference beam, and *I*_o_ and θ_o_(*x*, *y*) are the intensity and phase of the object beam after reflection/transmission by the object.

For the sake of clarity, let us consider quantum holography with undetected light in an induced coherence scenario (see Fig. 1B), where reference and object beams are generated by means of spontaneous parametric down-conversion (SPDC). The first important difference with classical holography is that the object beam after reflection/transmission from the object is not made to interfere with the reference beam. The object beam remains undetected. The second important difference for quantum holography is that the reference and object beam are incoherent beams. This can be seen from the fact that object and reference beam in the quantum holography scheme in principle have different spectra, which is indicated by the different colors used in Fig. 1B. If the transmission function of the object to be recorded is τ(*x*, *y*) = *t*(*x*, *y*)e^iθ(*x*, *y*)^ (∣τ∣ = *t*), then the spatially dependent degree of first-order coherence *g*^(1)^(*x*, *y*) between the two beams is *g*^(1)^(*x*, *y*) = *t*(*x*, *y*) (for further details, see the information in the Supplementary Materials).

In our work, we implement the two beams for the quantum holography scheme with undetected light by a photon pair source based on SPDC in nonlinear crystals. There, correlated signal and idler beams are generated. Arranging these two SPDC sources in a nonlinear SU(1,1) interferometer, signal and idler pairs are generated in either of the nonlinear crystals. Our experiments work in the low parametric gain regime, where the probability to generate signal-idler pairs in both nonlinear crystals simultaneously is negligible. In this regime, one can think that the hologram is the result of quantum interference between two possibilities characterized by the corresponding probability amplitudes. One possibility is that signal-idler pairs are generated in SPDC, the idler photons interact with the object and impinges on the second nonlinear crystal without inducing the emission of new paired photons. The second possibility is that signal-idler pairs are generated in the second nonlinear crystal, so no idler photons have traversed the object. The transmission function of the object determines the distinguishability of both possibilities and thus the degree of coherence of the two beams that interference in the hologram.

For quantum holography with undetected light, one finds (see Supplementary Materials for a detailed derivation) that the spatially dependent flux rate of signal photons detected *N*_Δφ_(*x*, *y*) when an object with transmission function *t*(*x*, *y*)e^iθ(*x*, *y*)^ is present in the idler path is (*24*).

$$N_{\Delta \varphi }(x,y)∼1+t(x,y)\text{cos}\;[\theta (x,y)-\nu (x,y)+\Delta \varphi ]$$ (2)

where Δφ is a global phases and ν(*x*, *y*) is a phase introduced by the nonlinear parametric down-conversion processes inside the nonlinear interferometer. This equation is slightly different from Eq. 1 that describes the interference pattern registered in classical holography. Nevertheless, both equations show that the amplitude and phase of light reflected/transmitted by an object can be registered in a medium (hologram) sensitive only to the intensity of light.

We aim at visualizing the interference pattern to be recorded in the hologram. For an unknown object, a single measurement does not allow to extract full phase [θ(*x*, *y*)] and amplitude [*t*(*x*, *y*)] information of the complex transmission coefficient introduced by the object. However, phase-shifting holography can be applied to extract this information, both amplitude and phase. In doing so, a series of images with global phases Δφ = 0, π/2, π,3π/2 can be recorded and processed. This will result in four images

$$\begin{array}{c} N_{0}(x,y)∼1+t(x,y)\text{cos}\;\theta (x,y)\\ N_{\pi /2}(x,y)∼1-t(x,y)\text{sin}\;\theta (x,y)\\ N_{\pi }(x,y)∼1-t(x,y)\text{cos}\;\theta (x,y)\\ N_{3\pi /2}(x,y)∼1+t(x,y)\text{sin}\;\theta (x,y) \end{array}$$ (3)

From these four measurements, one can easily extract the phase information

$$\theta (x,y)=\text{arctan}\;(\frac{N_{3\pi /2}-N_{\pi /2}}{N_{0}-N_{\pi }})$$ (4)

and the amplitude information

$$t(x,y)=2\times \frac{{\left\{{\left[N_{3\pi /2}-N_{\pi /2}\right]}^{2}+{\left[N_{0}-N_{\pi }\right]}^{2}\right\}}^{1/2}}{N_{0}+N_{\pi /2}+N_{\pi }+N_{3\pi /2}}$$ (5)

Recording four images of the spatially varying signal photon flux rate at a certain spectral range with a high efficiency detector allows retrieving full phase and amplitude information of an unknown object that is probed by photons in a different spectral range. In this way, quantum holography with undetected light becomes possible.

One can generalize this approach to a series of *M* ≥ 3 images (*34*). They need to be recorded with phases Δφ*_m_* = 2*m*π/*M* with *m* = 0…*M* − 1. Then, phase and amplitude information are given by

$$\theta (x,y)=-\text{arctan}\;(\frac{\sum_{m}N_{\Delta \varphi_{m}}(x,y)\text{sin}\;\Delta \varphi_{m}}{\sum_{m}N_{\Delta \varphi_{m}}(x,y)\text{cos}\;\Delta \varphi_{m}})$$ (6)

and

$$t(x,y)=2\times \frac{{\left\{{\left[\sum_{m}N_{\Delta \varphi_{m}}(x,y)\text{cos}\;\Delta \varphi_{m}\right]}^{2}+{\left[\sum_{m}N_{\Delta \varphi_{m}}(x,y)\text{sin}\;\Delta \varphi_{m}\right]}^{2}\right\}}^{1/2}}{\sum_{m}N_{\Delta \varphi_{m}}(x,y)}$$ (7)

One would expect that the consideration of a higher number of steps *M* will lead to a more accurate and precise reconstruction of the phase and amplitude, as it is the case in classical phase-shifting holography. Consequently, we have experimentally implemented quantum phase-shifting holography with undetected light for different step numbers. We have analyzed their impact on accuracy and the overall performance of each approach.

### Experimental setup

Most nonlinear interferometers implemented so far for quantum imaging and spectroscopy make use of one of two configurations. In one configuration, the signal photons generated in the first nonlinear crystal are detected and they never traverse the second nonlinear crystal (*6*, *20*, *29*). Only idler photons generated in the first nonlinear crystal, after being reflected/transmitted by the object impinge on the second nonlinear crystal. This is the original configuration put forward in 1991 by Mandel’s group (*20*, *35*).

In an alternative configuration, usually termed as SU(1,1) interferometer, the signal photons generated in the first nonlinear crystal are also injected in the second nonlinear crystal as the idler photons (*19*, *21*, *24*, *26*, *31*). Quantum interference explains the physical origin of the interference pattern for both configurations, and the mathematical expressions that describe the shape of this interference pattern are mostly equal. However, in the original configuration considered, we have three beams at the output (two signal beams and one idler beam), while in the SU(1,1) configuration, the output consists of two beams (signal and idler beams) (*31*).

**Fig. 2.**
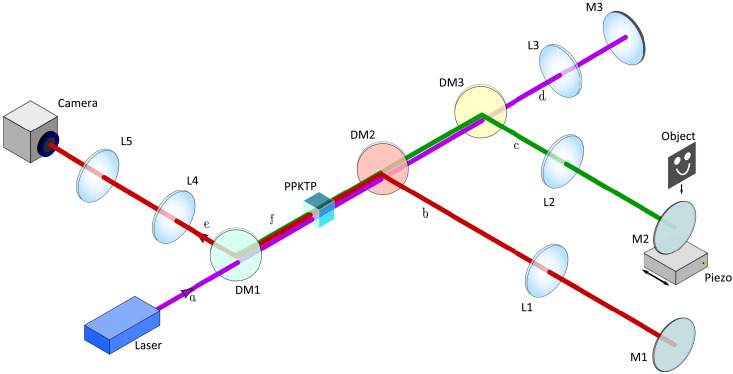
Experimental setup for holography with undetected light. Laser light (purple) pumps the nonlinear crystal (ppKTP) bidirectionally (beam paths a and d). It generates signal (red) and idler (green) beams either in the forward direction (beam paths b and c) or backward direction (beam paths e and f). Dichroic mirrors DM1 to DM3 separate the different beam paths. Idler light will illuminate the object (beam path c), while its hologram will be detected on the scientific complementary metal-oxide semiconductor (sCMOS) camera with the signal light (beam path e). The mirrors M1 to M3 are the interferometer end mirrors. M2 is mounted on a piezo stage to precisely move the mirror in one direction. Lenses L1 to L5 form the imaging system with the focal distances of 150 mm (L1, L2, and L3), 100 mm (L4), and 125 mm (L5).

Our experimental implementation of quantum holography with undetected light makes use of an SU(1,1) interferometer in a Michelson geometry as shown in Fig. 2. It consists of a nonlinear periodically poled potassium titanyl phosphate (ppKTP) crystal of 2 mm in length. The crystal is pumped bidirectionally with a collimated 405-nm continuous wave laser beam with up to 90 mW of pump power. Correlated signal and idler beams are emitted from the crystal via SPDC with central wavelengths of 910 and 730 nm, respectively. As shown in Fig. 2, the crystal is imaged onto itself with a 4f-system in each of the three interferometer arms via lenses L1 to L3. The object is placed in the Fourier plane of the idler arm having the momentum space at the object location. The interferometer end mirror M2 in the idler arm is mounted on a piezo translation stage (*36*) that allows to scan different phases of the interference produced by varying the path length. This way, one can define specific positions for the mirror M2 corresponding to different phase values Δφ. There is quantum interference between the two possibilities for the generation of signal-idler paired photons, after the pump, idler, and signal beams return to the crystal. In this way, the object’s information imprinted on the idler light is transferred to a spatially varying intensity of the signal light. Lenses L4 and L5 are used to form an image of the object at the camera plane. The camera (*37*) has 2048 × 2048 pixel with a pixel size of 6.5 μm. The images shown in this work are 500 × 500 pixel in size. The total usable field of view has a diameter of ≈ 6.1 mm.

### Holography and imaging performance

To experimentally test the quantum holography with undetected light approach, we used objects that were engraved in glass plates of refractive index 1.6 by grayscale lithography. The height of the engraved area was designed to induce a total phase change on the idler beam of either 0.62π or 0.82π, depending on the object. The dimensions of the objects and their features are shown in fig. S3.

**Fig. 3.**
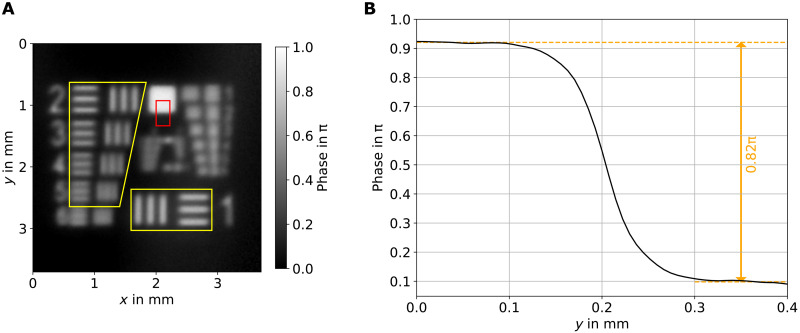
Hologram with undetected light of a resolution target. (**A**) Wide-field holographic image (using 12 frames) of the miniaturized resolution target, with a phase step of 0.82π (at the illumination wavelength). The elements contained in the yellow marked areas are the ones analyzed to determine the resolution of the setup (see Table 1). The red rectangle highlights the area used to verify the phase step of the object. (**B**) Phase step plot of the object, which matches with the manufactured 0.82π value.

Two of the objects are resolution targets that are a miniaturized version of the standard 1951 United States Air Force (USAF) resolution target to make all structures fit inside the field of view of the system (6.1 mm in diameter). One is with 0.62π phase step, and the other is with 0.82π. A full wide-field holographic image for the latter one can be seen in Fig. 3A. The area marked with a red rectangle is used to evaluate the phase step. As exemplarily shown in Fig. 3B, the desired phase step of 0.82π is well matched.

**Fig. 4.**
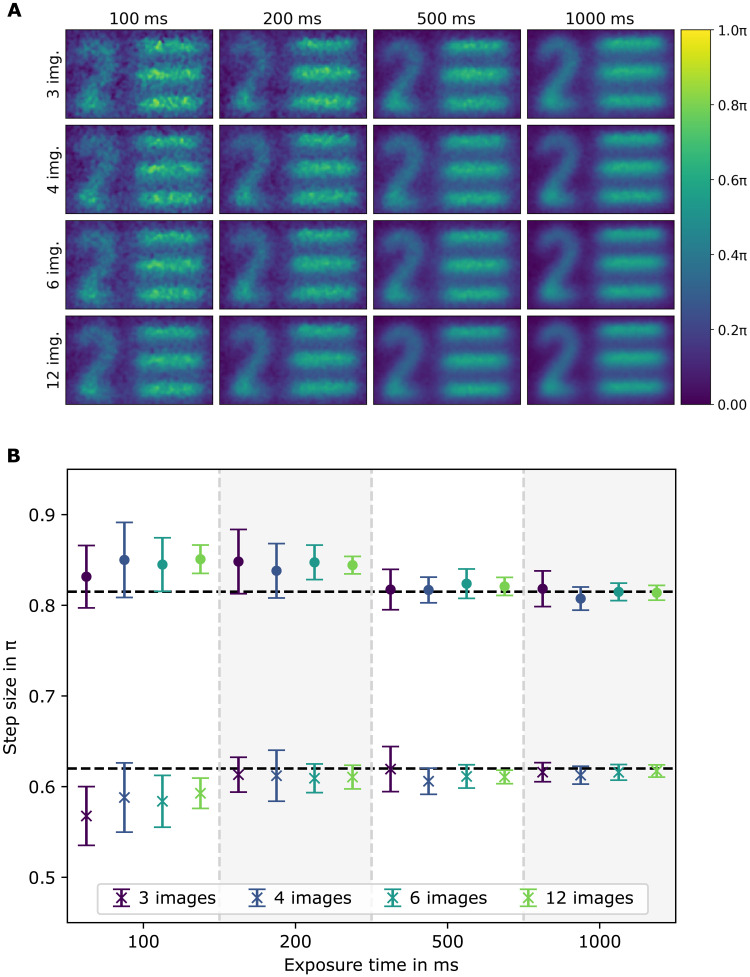
Phase accuracy. (**A**) Images of the same structure for different acquisition times of the camera and different number *M* of images used for the phase-shifting holography. (**B**) Calculated phase steps. Each point is an average of 15 image sets. The black dashed lines are the expected results. The color is in relation to the amount of images used (see legend). The cross markers refer to a sample with a step size of 0.62π, and the bullet markers refer to a sample with 0.82π step.

With both resolution objects, we investigated the impact of the number of images *M* recorded for the phase-shifting holography and the acquisition time per shot on a resolution target. In doing so, we varied the number of images from 3 to 12 and the acquisition time per image from 100 ms to 1 s. The results are shown in Fig. 4. A four-by-four image matrix shows the region of interest assigned to the “bar pattern 2” (Fig. 3) under different measurement settings. It clearly indicates that a higher number of images for the phase-shifting holography and a higher acquisition time lead to a better image quality. A quantitative evaluation is displayed in Fig. 4B for both resolution targets. The results of the phase step retrieval show that the calculated phase values match the expected values for exposure times higher than 200 ms. An increase in the amount of images or exposure time lowers the SD of the results due to a decrease of the measured noise (see fig. S4). According to these results, the current setup should be operated with 500-ms exposure time to ensure that the measured values align with the expected values within the SD. It is possible to do so with only *M* = 3 images, but *M* = 4 images lead to significant more accuracy. This results in at least 2-s overall measurement time for one phase-shifting holography measurement, disregarding the time needed to change the phase position and the postprocessing of the data. For this case, the maximum phase noise was measured to be (0.091 ± 0.005)π, which is the lowest detectable phase difference. The transmission of the object was measured to be homogeneous with *t* = (94 ± 1)% as in agreement with the theory taking into account the visibility with and without object (*24*).

The blurring in the phase images shows that the spatial resolution is visibly limited. Using the yellow marked bar patterns on the resolution target (see Fig. 3A), the contrast for different spatial frequencies can be calculated. It must be considered that it is a miniaturized version of the standard USAF target. Table 1 shows the sizes of the bar patterns and the measured contrast. Using the Rayleigh criterion, the measured contrast must be at least 14.2% to count as resolvable. Because the contrast is measured using bar patterns, which have multiple frequency components instead of sine patterns, it must be corrected by multiplying it with π/4 (*38*). These corrected contrast values are shown in the last two columns, named as sine wave contrast. The lowest measured resolvable spatial frequency according to the Rayleigh criteria is 6.3 mm^−1^, which corresponds to 79-μm feature size.

**Table 1.** Spatial resolution. Calculated contrast using the bar patterns of the miniaturized resolution target (yellow marked area in Fig. 3) for 1000-ms acquisition time and 0.82π phase step. Square wave contrast is the measured contrast on the bar pattern, and sine wave contrast is the corrected result, taken into account that the bar pattern contains multiple frequencies. The Line pair sizes marked with an asterisk (*) do not fulfill the Rayleigh criterion.

**Line pair**	**Frequency**	**Square wave contrast**		**Sine wave contrast**	
**in millimeter**	**in 1/mm**	**Vertical**	**Horizontal**	**Vertical**	**Horizontal**
0.200	5.0	0.60 ± 0.06	0.62 ± 0.06	0.47 ± 0.06	0.48 ± 0.06
0.178	5.6	0.53 ± 0.07	0.45 ± 0.08	0.42 ± 0.06	0.35 ± 0.07
0.158	6.3	0.36 ± 0.09	0.28 ± 0.09	0.29 ± 0.08	0.22 ± 0.08
0.140*	7.1	0.18 ± 0.10	0.12 ± 0.10	0.14 ± 0.09	0.09 ± 0.09
0.125*	8.0	0.07 ± 0.10	–	0.06 ± 0.09	–

**Fig. 5.**
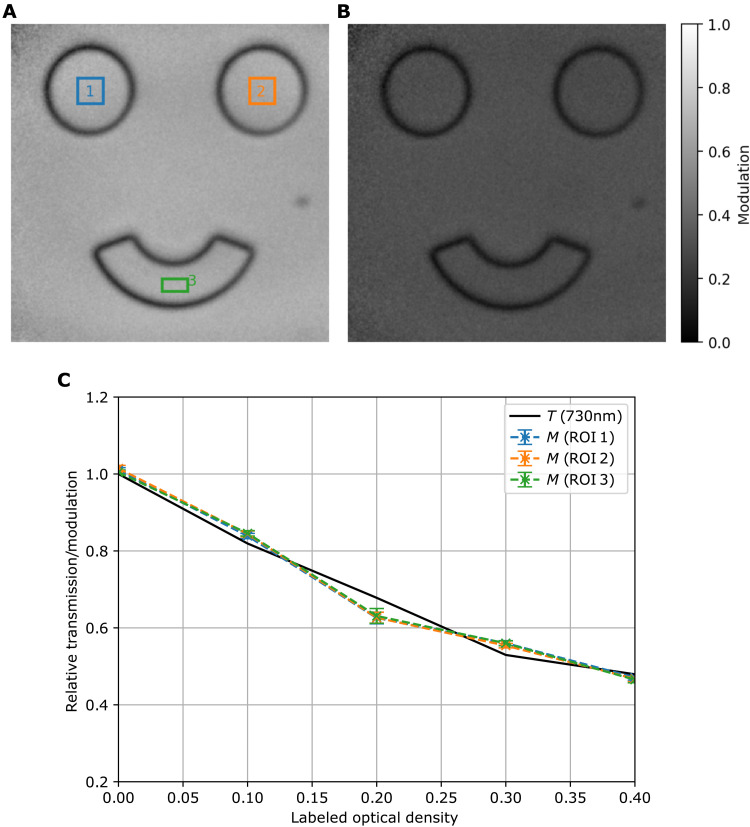
Amplitude accuracy. (**A**) Modulation image of a 0.82π phase step mask without OD filters. (**B**) Modulation image of the same mask fully covered with an OD filter (OD 0.4). (**C**) Relative modulation calculated values for the three different areas marked in the correspondent colors in (A) and expected transmission (black solid line) for different object transmission values, experimentally implemented by placing different OD filters in front of the phase mask. The modulation values are retrieved from holographic images with *M* = 12 phase steps each with 500-ms exposure time. ROI, region of interest.

In addition, we analyzed the accuracy of detecting transmission values. The transmission information can be obtained from the modulation of the interference pattern. In Materials and Methods, it is explained how the modulation can be obtained from the sampled images. While doing so, we recorded phase-shifting holograms with undetected light of an object in form of a happy face with different (but homogeneous) transmission values introduced by optical density (OD) filters. An example for the obtained modulation image is shown in Fig. 5 (A and B) for OD 0 and OD 0.4, respectively. The modulation of the interference and the transmission of the object follow a proportional relationship (*24*). The colored rectangles in Fig. 5A mark three regions of interest used to verify the homogeneity of the calculated object transmission across different areas of the field of view. The dependency of the transmission on the inserted OD filters for the three areas can be found in Fig. 5C and agrees well with the theoretical prediction. In fig. S5, one finds the uncertainty that is to be expected for a single measurement. For an exposure time of 500 ms and *M* = 4 images used, one gets a noise level of 0.053 ± 0.005, which means the setup can detect a minimum transmission difference of 5 to 6%. The visible border around the eyes and mouth of the object are due to the limited spatial resolution (see Supplementary Materials for a detailed explanation).

## DISCUSSION

We have introduced a novel technique to generate holograms with photons that are never detected. Our method is a quantum version of phase-shifting holography, which retrieves full information, phase, and amplitude of an object. To do so, we make use of correlated signal and idler photon pairs, in a nonlinear interferometer configuration to obtain a hologram of the idler photon, through the measurement of the signal photon. Our technique is based on the quantum effect of “induced coherence without induced emission,” where information imprinted on the undetected (idler) photon can be retrieved by analyzing the interference pattern of its partner (signal) photon. The main advantage of our technique over previous approaches is that we retrieved simultaneously the phase and amplitude of the shape of a photon despite never detecting this photon.

Our approach also alleviates a key challenge toward integrating SU(1,1) interferometers in real-world biomedical applications: Previous demonstrations of imaging with undetected photons required long-term phase stability of relevant paths in an interferometer and were thus either limited to operation in a controlled laboratory environment (*6*) or involved the added technical complexity of active stabilization. Our approach has reduced the required level phase stability to the order of 2 s (the time scale over which a complete round of phase settings can be applied). These levels of stability are straightforward to achieve in ruggedized optical assemblies (*24*)—a key advancement toward practical deployment.

We quantify our method in terms of the achievable resolution given by the number of measurements and the acquisition time. It turns out that a combination of 500-ms acquisition time and four phase-shifted images are an ideal combination in terms of recording time and image quality. We retrieve transmission values with a precision of down to 6% and phases with a precision of down to 0.1π. Increasing the degree of spatial correlation between signal and idler and the numerical aperture of the imaging system can markedly enhance the spatial resolution of this method. Moreover, by combining our technique with quantum optical coherence tomography in nonlinear interferometers (*26*, *29*–*31*), three-dimensional (3D) image reconstruction with undetected light becomes feasible.

## MATERIALS AND METHODS

### Image processing

**Fig. 6.**
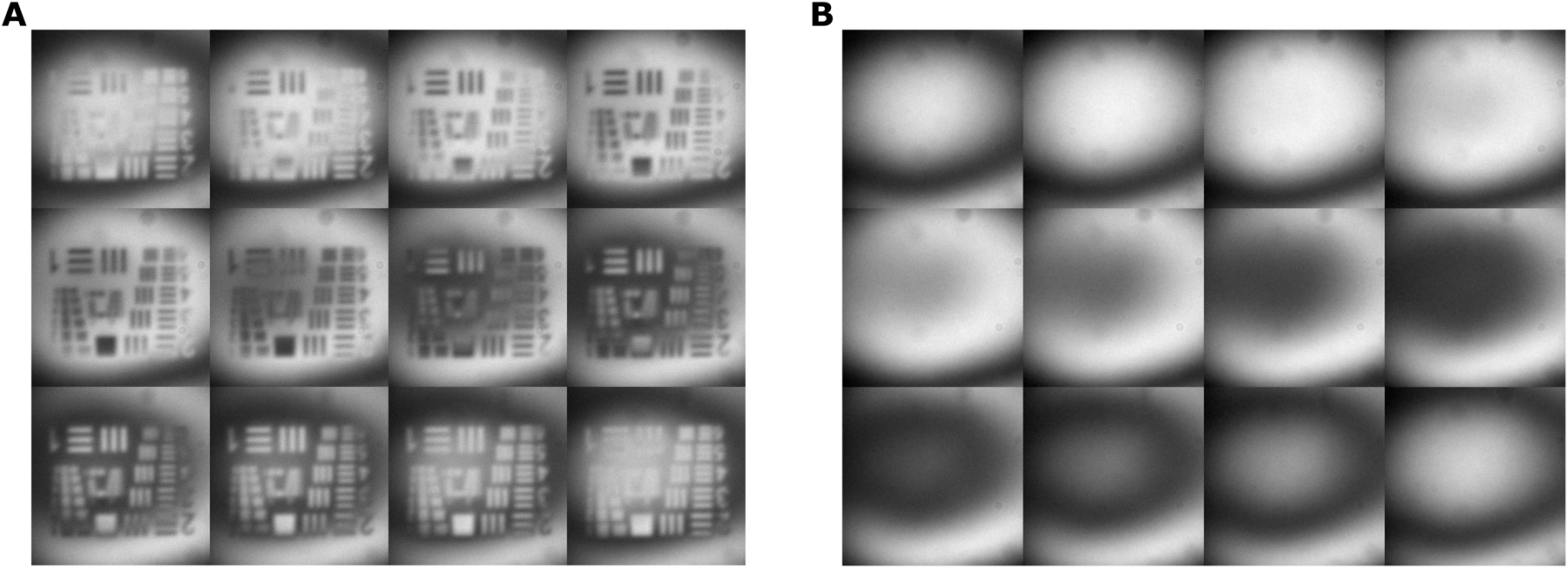
Exemplary raw image set. One set of recorded images for the phase-shifting holography calculation ordered from left to right and top to bottom. (**A**) The object images. (**B**) The reference images.

For the phase-shifting holography calculation, a set of 12 images of the pure interference as reference and another set of 12 images including the object is recorded (see Fig. 6). To prepare the images for the calculation, they were filtered to reduce the noise level. The filtering has three substeps. First, the camera background is subtracted. As background, a recording without pump beam was taken. Second, a 2D low-pass frequency filter is applied performing a Fourier transformation on the input images and cuts off high frequency noise. Last, a Gaussian filter is applied (*39*). With this, the signal-to-noise ratio was increased by a factor of up to 1.82 depending on the exposure time and number of images.

After the filtering, the phase and modulation are calculated. For this step, the images are separated into a subset depending on the algorithm to test. The base image set consists of 12 images taken at equally spaced phase positions with a phase difference of Δφ*_m_*= 2mπ/*M*; *m* = 0, …, *M* − 1 to the first position (*m* = 0). This set was split into four subset containing *M* = 12,6,4, and 3 images, equally spaced inside the 12 from the base set and always starting at the same image *m* = 0. This simulates measurements with less phase positions, without the need for additionally recordings to reduce differences between the measurements.

The calculation was done using a least squares–based algorithm for phase-shift holography (*34*). The phase is obtained by Eq. 6, with *N*_Δφ*_m_*_ as the recorded intensity of image *m*. A phase unwrapping algorithm is applied afterward to resolve the periodicity (*40*). After this, the phase of the reference is subtracted to get the absolute phase change induced by placing the object inside the setup. Our software gives real-time images (live feedback) for the expected modulation and phase. We introduced 300-ms delay time between each acquisition frame for the image postprocessing. The live feedback uses a constantly updated circular buffer for the phase positions. The code for the final postprocessed image needs approximately 45 s to evaluate 360 raw images, i.e., 15 measurement sets containing 12 raw images of the reference and the object. The results are 15 phase and modulation images. The calculation time can be substantially reduced implementing parallel processing and graphics processing unit. This is also true for the live feedback system.

In contrast to Eq. 7, losses at optical components will occur in the experiment. Hence, the interference modulation is measured, which is directly proportional to the transmission. Therefore, a referencing to an additional measurement without object is necessary to retrieve the absolute transmission values of an object. The modulation is calculated by Eq. 7.

### Object fabrication

The objects used in the experiments were generated by direct writing grayscale lithography. In doing so, a substrate is coated with a photoresist with a refractive index of 1.63 (at ∼520 nm) and exposed to ultraviolet light in a particular pattern. The chemical solubility of the exposed areas increases in alkaline medium, and the microstructure in the photoresist is formed.

## Supplementary Materials

### This PDF file includes:

Figs. S1 to S5 References
