# Supplementary material for: Quantum holography with undetected light
Source: Sci Adv. 2022 Jan 14;8(2):eabl4301. doi: 10.1126/sciadv.abl4301 (PMC8759747; doi:10.1126/sciadv.abl4301)
Supplement: Supplementary file 1 — Figs. S1 to S5 References [file sciadv.abl4301_sm.pdf]

Science Advances
AAAS

Supplementary Materials for

Quantum holography with undetected light

Sebastian Töpfer, Marta Gilaberte Basset, Jorge Fuenzalida, Fabian Steinlechner, Juan P. Torres, Markus Gräfe*

*Corresponding author. Email: markus.graefe@iof.fraunhofer.de

Published 14 January 2022, *Sci. Adv.* **8**, eabl4301 (2022)
DOI: 10.1126/sciadv.abl4301

This PDF file includes:

Figs. S1 to S5
References

Theory of Phase-shifting holography with a SU(1,1) nonlinear interferometer

General equations

The experimental set-up is depicted in Fig. 2 of the main text. The bandwidth (Δ_{DC}) of paired photons generated in parametric down-conversion (PDC) is much larger than the bandwidth of the pump beam (δ_p). The Rayleigh length ($L_R = k_p^0 w_p^2/2$) of the pump beam is much larger than the crystal length ($L \gg L_R$). w_p is the pump beam waist and $k_p^0 = 2\pi n_p/\lambda_p^0$ is the pump beam wavenumber. In this scenario we can describe the spatio-temporal characteristics of parametric down-conversion using the CW and plane pump beam approximation.

In the low parametric gain regime, the Bogoliuvov transformations that relate the quantum operators a_s and a_i at the output face of the nonlinear crystal to the quantum operators at the input face of the nonlinear crystal (b_s and b_i) are [42, 43]

$$a_s(\mathbf{q},\Omega) = U_s(\mathbf{q},\Omega)\, b_s(\mathbf{q},\Omega) + V_s(\mathbf{q},\Omega)\, b_i^\dagger(-\mathbf{q},-\Omega) \tag{S1}$$

$$a_i(\mathbf{q},\Omega) = U_i(\mathbf{q},\Omega)\, b_i(\mathbf{q},\Omega) + V_i(\mathbf{q},\Omega)\, b_s^\dagger(-\mathbf{q},-\Omega) \tag{S2}$$

where

$$\begin{aligned} U_{s,i}(\mathbf{q},\Omega) &= \exp\left[ik_{s,i}(\mathbf{q},\Omega)L\right] \\ V_{s,i}(\mathbf{q},\Omega) &= -i\,(\sigma L)\,\mathrm{sinc}\frac{\Delta_k L}{2}\exp\left\{i\left[k_p^0 + k_{s,i}(\mathbf{q},\Omega) - k_{i,s}(-\mathbf{q},-\Omega)\right]\frac{L}{2} + i\varphi_p\right\} \end{aligned} \tag{S3}$$

$\mathbf{q} = (q_x, q_y)$ designates the transverse wavenumber of signal/idler photons, Ω is the frequency deviation from the corresponding central frequency and φ_p is the phase of the pump beam. The nonlinear coefficient σ is

$$\sigma = \left(\frac{\hbar\omega_p\omega_s\omega_i[\chi^{(2)}]^2 F_0}{8\epsilon_0 c^3 S n_p n_s n_i}\right)^{1/2}. \tag{S4}$$

$F_0 = P_0/(\hbar\omega_p)$ is the flux rate density of pump photons (photons/s), S is the area of the pump beam and P_0 is the pump power. The low parametric gain regime corresponds to a gain $G = \sigma L \ll 1$.

The signal and idler beams fulfill the paraxial approximation, so we can expand the corresponding wavenumbers in a Taylor series as $k_{s,i} = k_{s,i}^0 + D_s\Omega - |\mathbf{q}|^2/(2k_{s,i}^0)$. $D_{s,i}$

are inverse group velocities. We assume phase matching at the central frequencies, i.e., $k_p^0 = k_s^0 + k_i^0 \pm 2\pi/\Lambda$ where Λ is the period of the poling of the nonlinear crystal. The phase matching function is thus

$$\Delta_k = D\Omega L - |\mathbf{q}|^2 \left[\frac{1}{2k_s^0} + \frac{1}{2k_i^0} \right] \tag{S5}$$

with $D = D_s - D_i$. Under these conditions, the functions $U_{s,i}$ and $V_{s,i}$ are invariant under changes $q_{x,y} \Longrightarrow -q_{x,y}$, a feature that we will use later on to simplify the expressions obtained.

In the first pass by the nonlinear crystal signal (s_1) and idler (i_1) beams are generated (beam paths b and c, respectively, in Fig. 2 of the main text). The corresponding quantum operators are $a_{s_1}(\mathbf{q}, \Omega)$ and $a_{i_1}(\mathbf{q}, \Omega)$. The signal beam traverses a 4f system with focal length f with a mirror located in the middle of the imaging system. After traversing this system the signal beam is injected back into the nonlinear crystal. There might be losses r_s in the signal path. The transformation of the quantum operator a_{s_1} is

$$a_{s_1}(q_x, q_y, \Omega) \Longrightarrow r_s a_{s_1}(q_x, -q_y, \Omega) \exp(i\varphi_s) + f_s \tag{S6}$$

with $\varphi_s = 4k_s^0 f + \Delta\varphi$. $\Delta\varphi$ is a phase that introduces a phase difference between signal and idler beam paths and that will be important to perform Phase shifting digital holography. The operators f_s, that fulfill the commutation relationship $[f_s, f_s^\dagger] = [1 - |r_s|^2]$, take into account losses in the signal path [44]. Notice that the transformation $q_y \longrightarrow -q_y$ in Eq. (S6), that only affects the y component of the wavenumber, is due to the peculiar 4f system that we are considering. It includes back-propagation caused by the presence of a mirror, which makes it different from other commonly considered 4f systems.

The idler beam i_1 traverses a 4f system with focal length f with an object with transmission function $t(\mathbf{x}) = |t(\mathbf{x})| \exp[i\theta(\mathbf{x})]$ in the middle. The transformation of the quantum operator a_{i_1} is

$$a_{i_1}(q_x, q_y, \Omega) \Longrightarrow t\left(\frac{\lambda_i f}{2\pi} q_x, -\frac{\lambda_i f}{2\pi} q_y \right) a_{i_1}(q_x, -q_y, \Omega) \exp(i\varphi_i) + f\left(\frac{\lambda_i f}{2\pi} q_x, -\frac{\lambda_i f}{2\pi} q_y \right) \tag{S7}$$

The phase is $\varphi_i = 4k_i^0 f$. The operators $f(\mathbf{q})$, that fulfill the commutation relationship

$$[f(\mathbf{q}), f^\dagger(\mathbf{q}')] = \left[1 - |t(\mathbf{q})|^2\right] \delta(\mathbf{q} - \mathbf{q}'), \tag{S8}$$

take into account the spatially-dependent losses induced by the transmission function.

The quantum operator that describes the signal beam s_2 is:

$$
\begin{aligned}
& a_{s_2}(q_x, q_y, \Omega) = r_s U_{s_2}(\mathbf{q}, \Omega) a_{s_1}(q_x, -q_y, \Omega) \exp(i\varphi_s) + U_{s_2}(\mathbf{q}, \Omega) f_s \\
& + V_{s_2}(\mathbf{q}, \Omega) t^* \left(-\frac{\lambda_i f}{2\pi} q_x, \frac{\lambda_i f}{2\pi} q_y \right) a_{i_1}^\dagger(-q_x, q_y, -\Omega) \exp(-i\varphi_i) \\
& + V_{s_2}(\mathbf{q}, \Omega) f^\dagger \left(-\frac{\lambda_i f}{2\pi} q_x, \frac{\lambda_i f}{2\pi} q_y \right) \\
& = r_s U_{s_2}(\mathbf{q}, \Omega) \left[U_{s_1}(\mathbf{q}, \Omega) b_s(q_x, -q_y, \Omega) + V_{s_1}(\mathbf{q}, \Omega) b_i^\dagger(-q_x, q_y, -\Omega) \right] \exp(i\varphi_s) \\
& + V_{s_2}(\mathbf{q}, \Omega) t^* \left(-\frac{\lambda_i f}{2\pi} q_x, \frac{\lambda_i f}{2\pi} q_y \right) \\
& \times \left[U_{i_1}^*(\mathbf{q}, -\Omega) b_i^\dagger(-q_x, q_y, -\Omega) + V_{i_1}^*(\mathbf{q}, -\Omega) b_s(q_x, -q_y, \Omega) \right] \exp(-i\varphi_i) \\
& + V_{s_2}(\mathbf{q}) f^\dagger \left(-\frac{\lambda_i f}{2\pi} q_x, \frac{\lambda_i f}{2\pi} q_y \right) + U_{s_2}(\mathbf{q}, \Omega) f_s \\
& = [r_s U_{s_2}(\mathbf{q}, \Omega) U_{s_1}(\mathbf{q}, \Omega) \exp(i\varphi_s) \\
& \left. + V_{s_2}(\mathbf{q}, \Omega) V_{i_1}^*(\mathbf{q}, -\Omega) t^* \left(-\frac{\lambda_i f}{2\pi} q_x, \frac{\lambda_i f}{2\pi} q_y \right) \exp(-i\varphi_i) \right] b_s(q_x, -q_y, \Omega) \\
& + [r_s U_{s_2}(\mathbf{q}, \Omega) V_{s_1}(\mathbf{q}, \Omega) \exp(i\varphi_s) \\
& \left. + U_{i_1}^*(\mathbf{q}, -\Omega) V_{s_2}(\mathbf{q}, \Omega) t^* \left(-\frac{\lambda_i f}{2\pi} q_x, \frac{\lambda_i f}{2\pi} q_y \right) \exp(-i\varphi_i) \right] b_i^\dagger(-q_x, q_y, -\Omega) \\
& + V_{s_2}(\mathbf{q}, \Omega) f^\dagger \left(-\frac{\lambda_i f}{2\pi} q_x, \frac{\lambda_i f}{2\pi} q_y \right) + U_{s_2}(\mathbf{q}, \Omega) f_s \; . \qquad \text{(S9)}
\end{aligned}
$$

Spatial shape of the flux rate of signal photons s_2

We measure the spatial distribution of the photon flux density corresponding to the signal beam s_2 with the help of a 2f system with focal length f_d. The flux rate of signal photons s_2 detected at time t is

$$
\langle N(\mathbf{x}, t) \rangle = \int d\Omega_1 d\Omega_2 \exp\left[i(\Omega_1 - \Omega_2)t \right] \left\langle a_{s_2}^\dagger \left(\frac{2\pi}{\lambda_s f_d} \mathbf{x}, \Omega_1 \right) a_{s_2} \left(\frac{2\pi}{\lambda_s f_d} \mathbf{x}, \Omega_2 \right) \right\rangle
$$

After the 2f system we use an imaging system with magnification $M = -1$. We obtain

$$\langle N(\mathbf{x},t)\rangle = \left(\frac{2\pi}{\lambda_s f_d}\right)^2 \int d\Omega\, F(\Omega) \left|V_{s_1}\left(\frac{2\pi}{\lambda_s f_d}\mathbf{x},\Omega\right)\right|^2 \left\{1 - \left|\tau\left(-\frac{\lambda_i}{\lambda_s}\frac{f}{f_d}x, \frac{\lambda_i}{\lambda_s}\frac{f}{f_d}y\right)\right|^2\right.$$
$$+ \left|r_s U_{s_2}\left(\frac{2\pi}{\lambda_s f_d}\mathbf{x},\Omega\right)\exp(i\varphi_s + i\varphi_{p_1})\right.$$
$$\left.\left. + U_{i_1}^*\left(\frac{2\pi}{\lambda_s f_d}\mathbf{x},-\Omega\right)\tau^*\left(-\frac{\lambda_i}{\lambda_s}\frac{f}{f_d}x, \frac{\lambda_i}{\lambda_s}\frac{f}{f_d}y\right)\exp(-i\varphi_i + i\varphi_{p_2})\right|^2\right\}$$
$$= \left(\frac{2\pi}{\lambda_s f_d}\right)^2 \int d\Omega\, F(\Omega) \left|V_{s_2}\left(\frac{2\pi}{\lambda_s f_d}\mathbf{x},\Omega\right)\right|^2$$
$$\times\left\{1 + r_s^2 + r_s U_{s_2}\left(\frac{2\pi}{\lambda_s f_d}\mathbf{x},\Omega\right) U_{i_1}\left(\frac{2\pi}{\lambda_s f_d}\mathbf{x},-\Omega\right)\tau\left(-\frac{\lambda_i}{\lambda_s}\frac{f}{f_d}x, \frac{\lambda_i}{\lambda_s}\frac{f}{f_d}y\right)\right.$$
$$\times\exp(i\varphi_s + i\varphi_i + i\Delta\varphi_p) + r_s U_{s_2}^*\left(\frac{2\pi}{\lambda_s f_d}\mathbf{x},\Omega\right) U_{i_1}^*\left(\frac{2\pi}{\lambda_s f_d}\mathbf{x},-\Omega\right)$$
$$\left.\times\tau^*\left(-\frac{\lambda_i}{\lambda_s}\frac{f}{f_d}x, \frac{\lambda_i}{\lambda_s}\frac{f}{f_d}y\right)\exp(-i\varphi_s - i\varphi_i - i\Delta\varphi_p)\right\} \qquad \text{(S10)}$$

where $\Delta\varphi_p = \varphi_{p_1} - \varphi_{p_2}$. r_s designate losses in the signal path and we have included the effect of filtering by means of the function $F(\Omega)$. If we make use explicitly of the modulus and phase of the transmission coefficient $\tau(x,y) = |\tau(x,y)|\exp[i\theta(x,y)]$, we have

$$\langle N(\mathbf{x},t)\rangle = \left(\frac{2\pi}{\lambda_s f_d}\right)^2 \int d\Omega\, F(\Omega) \left|V_{s_1}\left(\frac{2\pi}{\lambda_s f_d}\mathbf{x},\Omega\right)\right|^2 \left\{1 + r_s^2 + 2r_s\left|\tau\left(-\frac{\lambda_i}{\lambda_s}\frac{f}{f_d}x, \frac{\lambda_i}{\lambda_s}\frac{f}{f_d}y\right)\right|\right.$$
$$\left.\times\cos\left[\varphi_0 + D\Omega L - \left(\frac{2\pi}{\lambda_s f_d}\right)^2 L\left(\frac{1}{2k_s^0} + \frac{1}{2k_i^0}\right)|\mathbf{x}|^2 + \theta\left(-\frac{\lambda_i}{\lambda_s}\frac{f}{f_d}x, \frac{\lambda_i}{\lambda_s}\frac{f}{f_d}y\right)\right]\right\} \qquad \text{(S11)}$$

where $\varphi_0 = \varphi_s + \varphi_i + \Delta\varphi_p + k_s^0 L + k_i^0 L$ is a constant phase.

Amplitude and phase retrieval with phase shifting holography

We consider the measurement of $\langle N(\mathbf{x},t)\rangle$ for four values of the reference phase, $\varphi_0 = 0, \pi/2, \pi, 3\pi/2$. Let us designate

$$\nu = \left(\frac{2\pi}{\lambda_s f_d}\right)^2 L\left(\frac{1}{2k_s^0} + \frac{1}{2k_i^0}\right) = \frac{\pi L}{(\lambda_s f_d)^2}\left(\frac{\lambda_s}{n_s} + \frac{\lambda_i}{n_i}\right) \qquad \text{(S12)}$$

and $r_0 = 1 + r_s^2$. We can write

$$N_0(\mathbf{x}) = \alpha \int d\Omega F(\Omega) \left| V_{s_1}\left(\frac{2\pi}{\lambda_s f}\mathbf{x}, \Omega\right)\right|^2 \tag{S13}$$
$$\times \left\{ r_0 + 2r_s \left|\tau\left(-\frac{\lambda_i}{\lambda_s}\frac{f}{f_d}x, \frac{\lambda_i}{\lambda_s}\frac{f}{f_d}y\right)\right| \cos\left[\theta\left(-\frac{\lambda_i}{\lambda_s}\frac{f}{f_d}x, \frac{\lambda_i}{\lambda_s}\frac{f}{f_d}y\right) + D\Omega L - \nu|\mathbf{x}|^2\right]\right\}$$
$$N_{\pi/2}(\mathbf{x}) = \alpha \int d\Omega F(\Omega) \left| V_{s_1}\left(\frac{2\pi}{\lambda_s f}\mathbf{x}, \Omega\right)\right|^2 \tag{S14}$$
$$\times \left\{ r_0 - 2r_s \left|\tau\left(-\frac{\lambda_i}{\lambda_s}\frac{f}{f_d}x, \frac{\lambda_i}{\lambda_s}\frac{f}{f_d}y\right)\right| \sin\left[\theta\left(-\frac{\lambda_i}{\lambda_s}\frac{f}{f_d}x, \frac{\lambda_i}{\lambda_s}\frac{f}{f_d}y\right) + D\Omega L - \nu|\mathbf{x}|^2\right]\right\}$$
$$N_{\pi}(\mathbf{x}) = \alpha \int d\Omega F(\Omega) \left| V_{s_1}\left(\frac{2\pi}{\lambda_s f}\mathbf{x}, \Omega\right)\right|^2 \tag{S15}$$
$$\times \left\{ r_0 - 2r_s \left|\tau\left(-\frac{\lambda_i}{\lambda_s}\frac{f}{f_d}x, \frac{\lambda_i}{\lambda_s}\frac{f}{f_d}y\right)\right| \cos\left[\theta\left(-\frac{\lambda_i}{\lambda_s}\frac{f}{f_d}x, \frac{\lambda_i}{\lambda_s}\frac{f}{f_d}y\right) + D\Omega L - \nu|\mathbf{x}|^2\right]\right\}$$
$$N_{3\pi/2}(\mathbf{x}) = \alpha \int d\Omega F(\Omega) \left| V_{s_1}\left(\frac{2\pi}{\lambda_s f}\mathbf{x}, \Omega\right)\right|^2 \tag{S16}$$
$$\times \left\{ r_0 + 2r_s \left|\tau\left(-\frac{\lambda_i}{\lambda_s}\frac{f}{f_d}x, \frac{\lambda_i}{\lambda_s}\frac{f}{f_d}y\right)\right| \sin\left[\theta\left(-\frac{\lambda_i}{\lambda_s}\frac{f}{f_d}x, \frac{\lambda_i}{\lambda_s}\frac{f}{f_d}y\right) + D\Omega L - \nu|\mathbf{x}|^2\right]\right\}$$

where α is a constant that depends on the detection efficiency and $F(\Omega)$ describes the frequency response of a frequency filter.

The phase of the transmission function can be evaluated with

$$\theta\left(-\frac{\lambda_i}{\lambda_s}\frac{f}{f_d}x, \frac{\lambda_i}{\lambda_s}\frac{f}{f_d}y\right) = \tan^{-1}\frac{N_{3\pi/2}(\mathbf{x}) - N_{\pi/2}(\mathbf{x})}{N_0(\mathbf{x}) - N_{\pi}(\mathbf{x})} + \nu|\mathbf{x}|^2 \tag{S17}$$

We can get rid of the phase induced by the parametric process, i.e. $\nu|\mathbf{x}|^2$, by subtracting the phases calculated with and without object present.

The amplitude can be evaluated by

$$\left|\tau\left(-\frac{\lambda_i}{\lambda_s}\frac{f}{f_d}x, \frac{\lambda_i}{\lambda_s}\frac{f}{f_d}y\right)\right| = \frac{1+r_s^2}{r_s}\,\frac{\left\{[N_0(\mathbf{x}) - N_{\pi}(\mathbf{x})]^2 + \left[N_{\pi/2}(\mathbf{x}) - N_{3\pi/2}(\mathbf{x})\right]^2\right\}^{1/2}}{N_0(\mathbf{x}) + N_{\pi/2}(\mathbf{x}) + N_{\pi}(\mathbf{x}) + N_{3\pi/2}(\mathbf{x})} \tag{S18}$$

We are not considering that pixels have a finite size S centered around the center position $\mathbf{x}_0$ of each pixel. We can take into account this effect making the substitutions $N_{\Delta\varphi}(\mathbf{x}_0) \implies \int_S d\mathbf{x} N_{\Delta\varphi}(\mathbf{x})$. As a result images of sharp edges will be seen as smoother transitions.

Effect of the bandwidth of PDC in amplitude and phase retrieval: analytical approximation

To help understanding the effect of the bandwidth of PDC in phase shifting holography, we notice that Eqs. (S14), (S15), (S16) and (S17) can be well approximated as

$$I = r_0 \int d\Omega \exp\left[-\frac{\Omega^2}{B_f^2} - \gamma^2 \left(\frac{D\Omega L}{2} - \frac{\nu|\mathbf{x}|^2}{2}\right)^2\right]$$
$$+2r_s|t| \int d\Omega \exp\left[-\frac{\Omega^2}{B_f^2} - \gamma^2 \left(\frac{D\Omega L}{2} - \frac{\nu|\mathbf{x}|^2}{2}\right)^2\right] \cos\left[D\Omega L + \varphi_0 - \nu|\mathbf{x}|^2 + \theta\right] \quad \text{(S19)}$$

We have assumed Gaussian filtering $F(\Omega) = \exp(-\Omega^2/B_f^2)$ and we have approximated the sinc function by an exponential: $\text{sinc}^2\, x \sim \exp(-\gamma^2 x^2)$ with the same FWHM ($\gamma^2 = 0.3588$). The bandwidth of PDC is $B_{dc} = 4/[\gamma^2(DL)^2]$.

These integrals have analytical expressions and yield

$$I = \left(\frac{4\pi B_f^2}{4+\gamma^2(DL)^2B_f^2}\right)^{1/2} \exp\left\{-\frac{\gamma^2\nu^2|\mathbf{x}|^4}{4} + \frac{4B_f^2}{4+\gamma^2(DL)^2B_f^2}\frac{\gamma^4(DL)^2\nu^2|\mathbf{x}|^4}{16}\right\} \quad \text{(S20)}$$
$$+\left\{r_0 + 2r_s|t| \exp\left(-\frac{B_f^2(DL)^2}{4+\gamma^2(DL)^2B_f^2}\right) \cos\left[\varphi_0 + \theta - \nu|\mathbf{x}|^2 \left(1 - \frac{\gamma^2(DL)^2B_f^2}{4+\gamma^2(DL)^2B_f^2}\right)\right]\right\}$$

Two main conclusions can be drawn from Eq. (S20). The first conclusion is that the bandwidth of PDC makes that the amplitude measured with phase shifting holography is not the real amplitude of the object ($|t|$) but an effective value $|t|_{eff} = |t| \exp\left(-\frac{B_f^2(DL)^2}{4+\gamma^2(DL)^2B_f^2}\right)$. For small bandwidths ($B_f \longrightarrow 0$) the correct amplitude $|t|$ is retrieved However, for larger bandwidth $|t|_{eff} \exp\left(-\frac{1}{\gamma^2}\right)$. Taking into account that

$$\int d\Omega \exp\left(-\frac{\gamma^2(DL)^2\Omega^2}{4}\right)[1+\cos(D\Omega L+\theta)] = \frac{\sqrt{4\pi}}{\gamma(DL)}\left[1+\exp\left(-\frac{1}{\gamma^2}\right)\cos\theta\right] \quad \text{(S21)}$$

we notice that the factor $\exp(-1/\gamma^2) = 0.06$ is the loss of visibility that one would observe in an interference experiment due to the finite bandwidth of the signal. We should highlight that using the exact solution with the sinc function we would have obtained 0.

The second conclusion is that the phase induced by the PDC process is modified, i.e., $\nu|\mathbf{x}|^2 \Longrightarrow \nu|\mathbf{x}|^2\left[1 - \left(\gamma^2(DL)^2B_f^2\right)/\left(4+\gamma^2(DL)^2B_f^2\right)\right]$. However when subtracting phases measured with and without the object, the phase of the object θ can still be correctly retrieved even for large bandwidths.

Influence of the spatial resolution on the interference amplitude

The modulation images shown in this paper have a visible modulation drop on places where a step-like phase change is located (see Fig. S1). This behaviour is caused by the limited spatial resolution. The interference patterns overlap partially in this areas, resulting in sum to a new interference with lower amplitude. Figure S2 shows this behaviour simulated with two interference patterns of the same base amplitude and phase-steps of the same value as our samples. The phase-step with limited resolution was simulated by shaping the base amplitudes as error-functions. The graph shows that we can expect amplitude drops to 28% for a 0.82π phase-step and 56% for a 0.62π phase-step in the ideal case. In the case of 0.82π we could measure a drop to 38% of the amplitude. The difference may be caused by measurement imperfections.

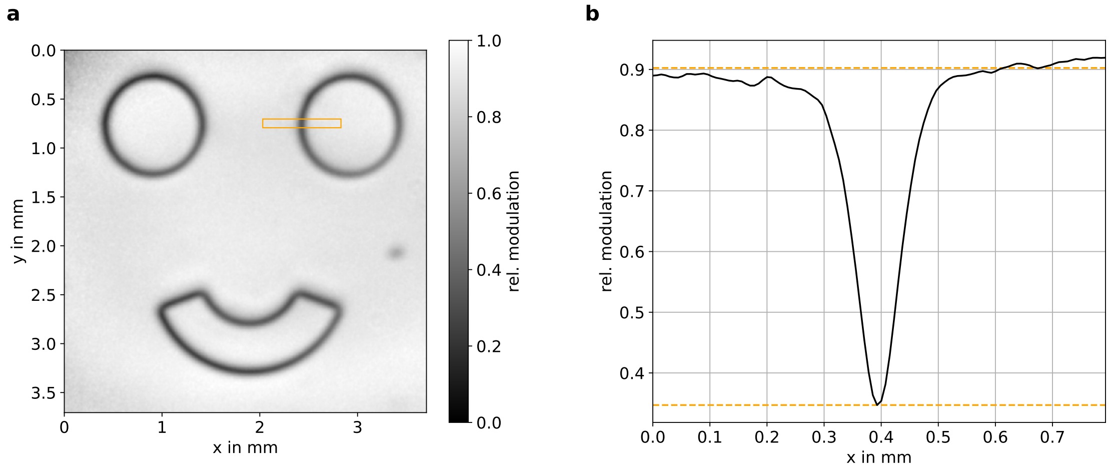

Figure S1: **Modulation drop profile.**(a) Modulation image of the happy face target with a phase-step of 0.82π. (b) Profile of the modulation in the marked are with visible drop due to interference amplitude decrease.

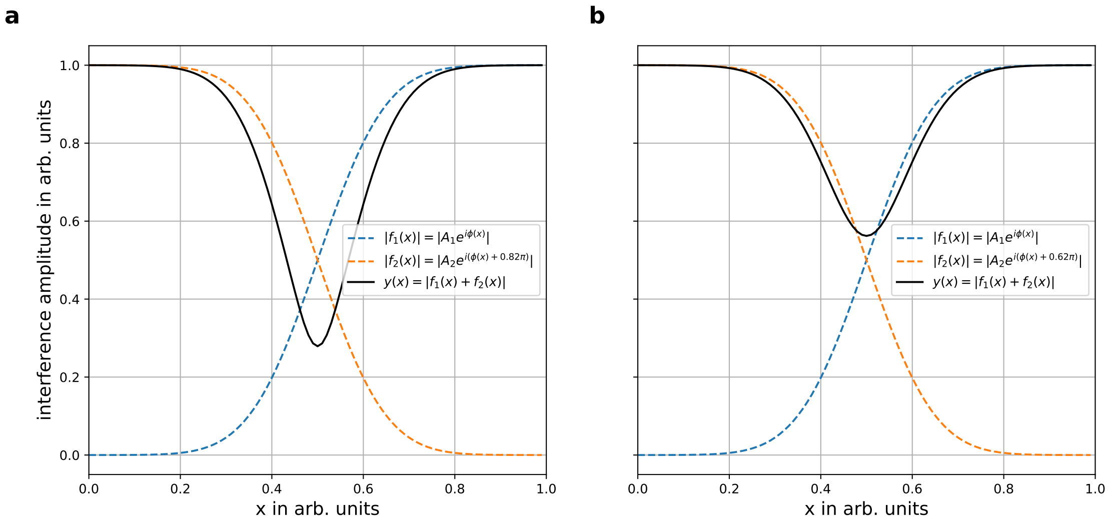

Figure S2: **Simulated interference amplitude drop**. The interference amplitude drops due to overlapping interference patterns, caused by limited spatial resolution for (a) 0.82π and (b) 0.62π phase difference.

Object dimensions

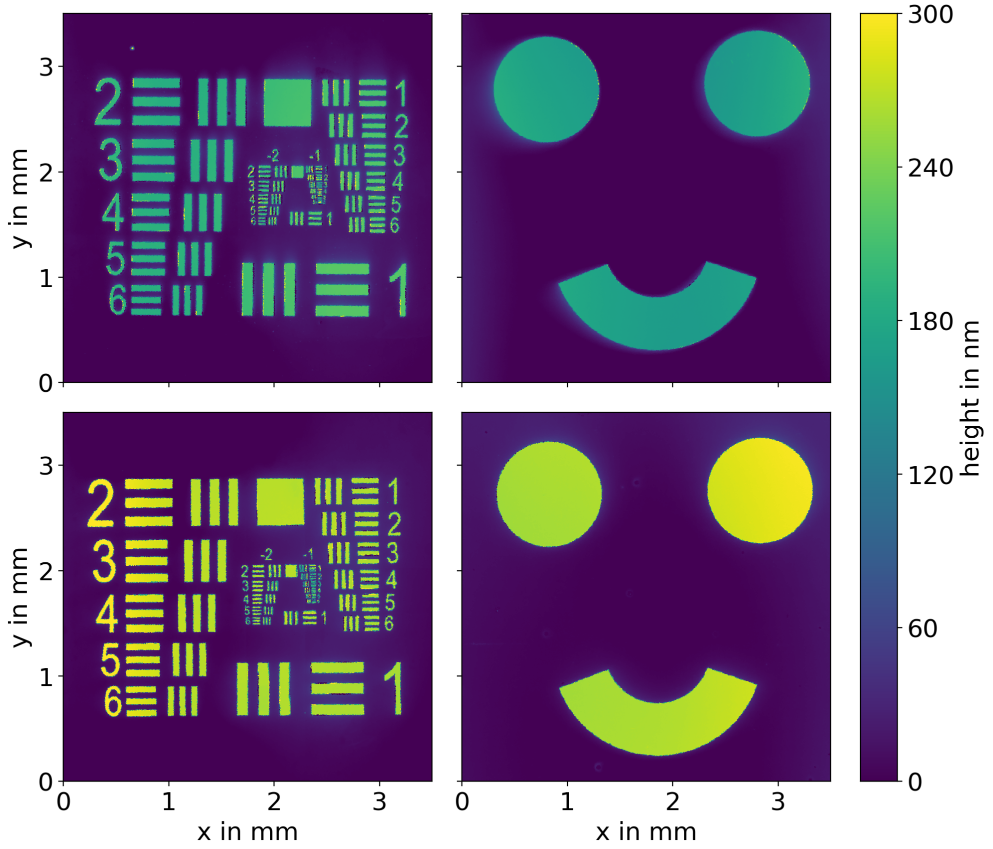

Figure S3: **Dimensions of the sample objects.** (top row) Miniaturized USAF and smiley phase targets manufactured to introduce a 0.62π phase shift. (bottom row) Miniaturized USAF and smiley phase targets manufactured to introduce a 0.82π phase shift. The phase shift introduced is calculated from the path difference seen for light at 730 nm travelling through the different sample thickness (height colorbar).

Measured phase and transmission noise

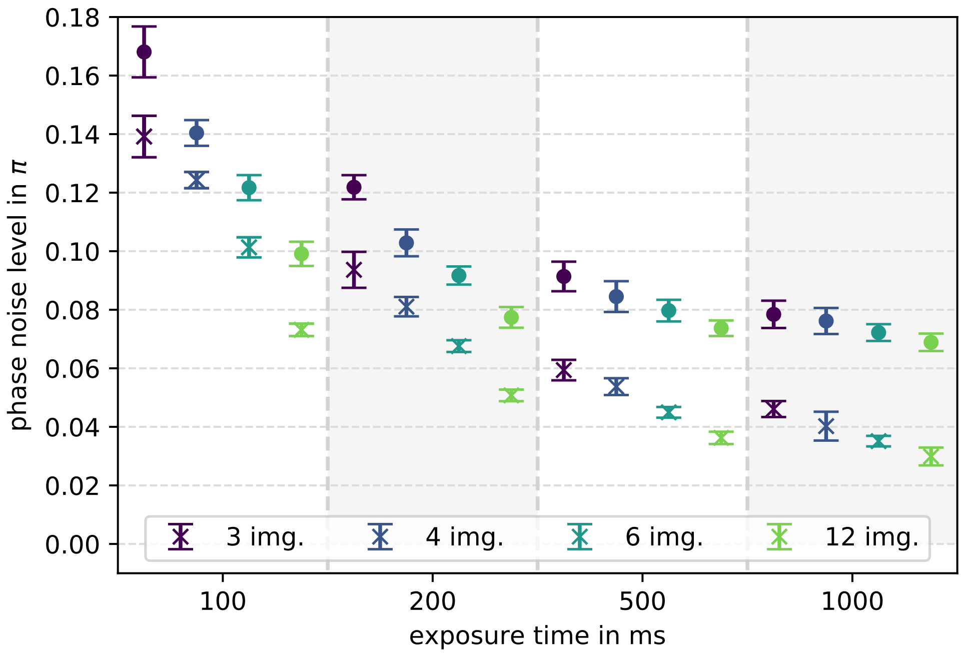

Figure S4: **Measured phase noise level.** The noise is measured as the standard deviation of the phase values at the high level of the phase step. Each points is an average of 15 image sets. The color is in relation to the amount of image used for the calculation of one phase image (see legend). The cross markers refer to a sample with a step size of 0.62π and the bullet markers refer to a sample with 0.82π step.

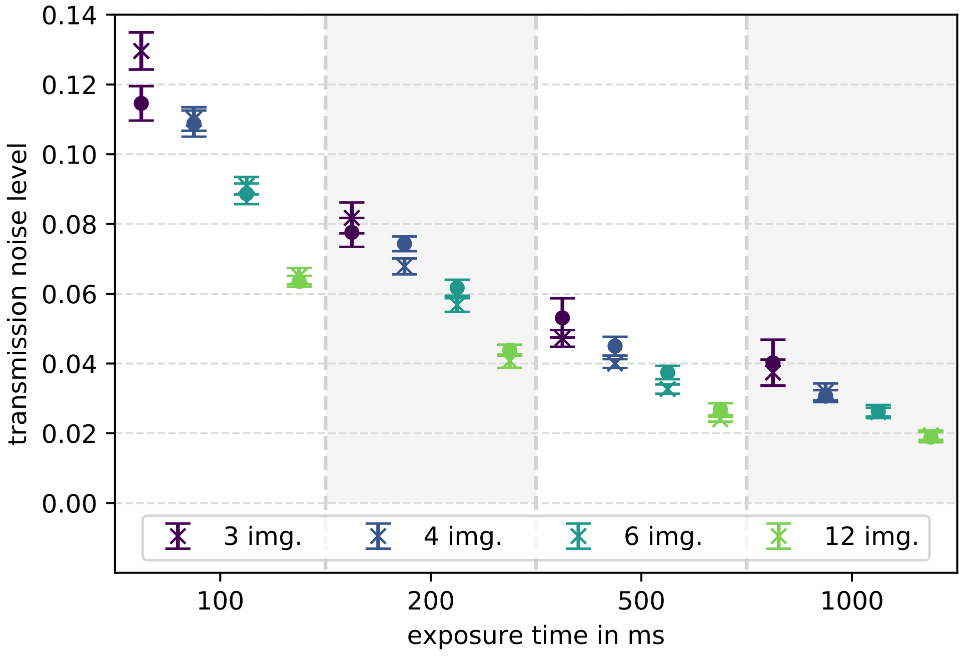

Figure S5: **Measured transmission noise level.** The noise is measured as the standard deviation of the transmission values. Each points is an average of 15 image sets. The color is in relation to the amount of image used for the calculation of one modulation image (see legend). The cross markers refer to a sample with a step size of 0.62π and the bullet markers refer to a sample with 0.82π step.

REFERENCES AND NOTES

1. F. Xu, X. Ma, Q. Zhang, H.-K. Lo, J.-W. Pan, Secure quantum key distribution with realistic devices. *Rev. Mod. Phys.* **92**, 025002 (2020).

2. S. Wengerowsky, J. Siddarth Koduru, F. Steinlechner, J. R. Zichi, B. Liu, T. Scheidl, S. M. Dobrovolskiy, R. van der Molen, J. W. N. Los, V. Zwiller, M. A. M. Versteegh, A. Mura, D. Calonico, M. Inguscio, A. Zeilinger, A. Xuereb, R. Ursin, Passively stable distribution of polarisation entanglement over 192 km of deployed optical fibre. *npj Quantum Inf.* **6**, 5 (2020).

3. E. Ortega, K. Dovzhik, J. Fuenzalida, S. Wengerowsky, J. C. Alvarado-Zacarias, R. F. Shiozaki, R. Amezcua-Correa, M. Bohmann, R. Ursin, Experimental space-division multiplexed polarization entanglement distribution through a 19-path multicore fiber. arXiv [preprint] arXiv:2103.10791 [quant-ph] (2021).

4. H.-S. Zhong, H. Wang, Y.-H. Deng, M.-C. Chen, L.-C. Peng, Y.-H. Luo, J. Qin, D. Wu, X. Ding, Y. Hu, P. Hu, X.-Y. Yang, W.-J. Zhang, H. Li, Y. Li, X. Jiang, L. Gan, G. Yang, L. You, Z. Wang, L. Li, N.-L. Liu, C.-Y. Lu, J.-W. Pan, Quantum computational advantage using photons. *Science* **370**, 1460–1463 (2020).

5. G. Brida, M. Genovese, I. R. Berchera, Experimental realization of sub-shot-noise quantum imaging. *Nat. Photonics* **4**, 227–230 (2010).

6. G. B. Lemos, V. Borish, G. D. Cole, S. Ramelow, R. Lapkiewicz, A. Zeilinger, Quantum imaging with undetected photons. *Nature* **512**, 409–412 (2014).

7. D. A. Kalashnikov, A. V. Paterova, S. P. Kulik, L. A. Krivitsky, Infrared spectroscopy with visible light. *Nat. Photonics* **10**, 98–101 (2016).

8. M. Gilaberte Basset, F. Setzpfandt, F. Steinlechner, E. Beckert, T. Pertsch, M. Gräfe, Perspectives for applications of quantum imaging. *Laser Photonics Rev.* **13**, 1900097 (2019).

9. P.-A. Moreau, E. Toninelli, T. Gregory, M. J. Padgett, Imaging with quantum states of light. *Nat. Rev. Phys.* **1**, 367–380 (2019).

10. H. Defienne, B. Ndagano, A. Lyons, D. Faccio, Polarization entanglement-enabled quantum holography. *Nat. Phys.* **17**, 591–597 (2021).

11. R. Chrapkiewicz, M. Jachura, K. Banaszek, W. Wasilewski, Hologram of a single photon. *Nat. Photonics* **10**, 576–579 (2016).

12. D. Gabor, A new microscopic principle. *Nature* **161**, 777–778 (1948).

13. G. Popescu, *Quantitative Phase Imaging of Cells and Tissues* (McGraw-Hill Education, 2011).

14. P. Marquet, B. Rappaz, P. J. Magistretti, E. Cuche, Y. Emery, T. Colomb, C. Depeursinge, Digital holographic microscopy: A noninvasive contrast imaging technique allowing quantitative visualization of living cells with subwavelength axial accuracy. *Opt. Lett.* **30**, 468–470 (2005).

15. P. Refregier, B. Javidi, Optical image encryption based on input plane and Fourier plane random encoding. *Opt. Lett.* **20**, 767–769 (1995).

16. S. Liu, C. Guo, J. T. Sheridan, A review of optical image encryption techniques. *Opt. Laser Technol.* **57**, 327–342 (2014).

17. F.-K. Bruder, R. Hagen, T. Rölle, M.-S. Weiser, T. Fäcke, From the surface to volume: Concepts for the next generation of optical-holographic data-storage materials. *Angew. Chem. Int. Ed.* **50**, 4552–4573 (2011).

18. W. L. Wilson, K. R. Curtis, K. E. Anderson, M. C. Tackitt, A. J. Hill, M. Pane, C. Stanhope, T. Earhart, W. Loechel, C. Bergman, K. Wolfgang, C. Shuman, G. Hertrich, K. Pharris, K. Malang, B. Riley, M. Ayres, Realization of high-performance holographic data storage: The InPhase Technologies demonstration platform, in *Organic Holographic Materials and Applications*, K. Meerholz, Ed. (International Society for Optics and Photonics, SPIE, 2003), vol. 5216, pp. 178–191.

19. B. Yurke, S. L. McCall, J. R. Klauder, SU(2) and SU(1,1) interferometers. *Phys. Rev. A* **33**, 4033–4054 (1986).

20. L. J. Wang, X. Y. Zou, L. Mandel, Induced coherence without induced emission. *Phys. Rev. A* **44**, 4614–4622 (1991).

21. T. J. Herzog, J. G. Rarity, H. Weinfurter, A. Zeilinger, Frustrated two-photon creation via interference. *Phys. Rev. Lett.* **72**, 629–632 (1994).

22. M. V. Chekhova, Z. Y. Ou, Nonlinear interferometers in quantum optics. *Adv. Opt. Photon.* **8**, 104 (2016).

23. A. C. Cardoso, L. P. Berruezo, D. F. Ávila, G. B. Lemos, W. M. Pimenta, C. H. Monken, P. L. Saldanha, S. Pádua, Classical imaging with undetected light. *Phys. Rev. A* **97**, 033827 (2018).

24. M. Gilaberte Basset, A. Hochrainer, S. Töpfer, F. Riexinger, P. Bickert, J. R. León-Torres, F. Steinlechner, M. Gräfe, Video-rate imaging with undetected photons. *Laser Photonics Rev.* **15**, 2000327 (2021).

25. M. Kutas, B. Haase, P. Bickert, F. Riexinger, D. Molter, G. von Freymann, Terahertz quantum sensing. *Sci. Adv.* **6**, eaaz8065 (2020).

26. A. V. Paterova, H. Yang, C. An, D. A. Kalashnikov, L. A. Krivitsky, Tunable optical coherence tomography in the infrared range using visible photons. *Quantum Sci. Technol.* **3**, 025008 (2018).

27. I. Kviatkovsky, H. M. Chrzanowski, E. G. Avery, H. Bartolomaeus, S. Ramelow, Microscopy with undetected photons in the mid-infrared. *Sci. Adv.* **6**, eabd0264 (2020).

28. A. V. Paterova, S. M. Maniam, H. Yang, G. Grenci, L. A. Krivitsky, Hyperspectral infrared microscopy with visible light. *Sci. Adv.* **6**, eabd0460 (2020).

29. A. Vallés, G. Jiménez, L. J. Salazar-Serrano, J. P. Torres, Optical sectioning in induced coherence tomography with frequency-entangled photons. *Phys. Rev. A* **97**, 023824 (2018).

30. A. Vanselow, P. Kaufmann, I. Zorin, B. Heise, H. M. Chrzanowski, S. Ramelow, Frequency-domain optical coherence tomography with undetected mid-infrared photons. *Optica* **7**, 1729 (2020).

31. G. J. Gerard J. Machado, G. Frascella, J. P. Torres, M. V. Chekhova, Optical coherence tomography with a nonlinear interferometer in the high parametric gain regime. *Appl. Phys. Lett.* **117**, 094002 (2020).

32. I. Yamaguchi, T. Zhang, Phase-shifting digital holography. *Opt. Lett.* **22**, 1268 (1997).

33. J. W. Goodman, *Introduction to Fourier Optics* (Roberts and Company Publishers, 2005).

34. H. Schreiber, J. H. Bruning, *Phase Shifting Interferometry* (John Wiley & Sons, Ltd, 2007), chap. 14, pp. 547–666.

35. X. Y. Zou, L. J. Wang, L. Mandel, Induced coherence and indistinguishability in optical interference. *Phys. Rev. Lett.* **67**, 318–321 (1991).

36. SmarAct GmbH, "Specifications: CLS-3232"; www.smaract.com/linear-stages/product/cls-3232-linear-stage-with-edu [accessed 25 August 2021].

37. Teledyne Photometrics, "Specifications: Prime BSI Scientific CMOS"; www.photometrics.com/products/prime-family/primebsi [accessed 25 August 2021].

38. N. B. Nill, "Conversion between sine wave and square wave spatial frequency response of an imaging system" (Technical report, MITRE, 2001).

39. "scipy.ndimage.gaussian−filter"; https://docs.scipy.org/doc/scipy/reference/generated/scipy.ndimage.gaussian_filter.html [accessed 13 December 2020].

40. "unwrap−phase"; https://scikit-image.org/docs/stable/api/skimage.restoration.html#kimage.restoration.unwrap_phase [accessed 13 December 2020].

41. A. Gatti, R. Zambrini, M. San Miguel, L. A. Lugiato, Multiphoton multimode polarization entanglement in parametric down-conversion. *Phys. Rev. A* **68**, 053807 (2003).

42. J. P. Torres, K. Banaszek, I. A. Walmsley, Engineering nonlinear optic sources of photonic entanglement. *Prog. Opt.* **56**, 227–331 (2011).

43. R. W. Boyd, G. S. Agarwal, K. W. C. Chan, A. K. Jha, M. N. O'Sullivan, Propagation of quantum states of light through absorbing and amplifying media. *Opt. Commun.* **281**, 3732–3738 (2008).
